# When a joint model should be preferred over a linear mixed model for analysis of longitudinal health-related quality of life data in cancer clinical trials

**DOI:** 10.1186/s12874-023-01846-3

**Published:** 2023-02-10

**Authors:** Célia Touraine, Benjamin Cuer, Thierry Conroy, Beata Juzyna, Sophie Gourgou, Caroline Mollevi

**Affiliations:** 1 Biometrics Unit, Cancer Institute of Montpellier, University of Montpellier, Montpellier, France; 2 French National Platform Quality of Life and Cancer, Montpellier, France; 3 Desbrest Institute of Epidemiology and Public Health, IDESP UMR UA11 INSERM, University of Montpellier, Montpellier, France; 4 Department of Medical Oncology, Institut de cancérologie de Lorraine, Vandoeuvre-lès-Nancy, France; 5 Team MICS, APEMAC, Université de Lorraine, Nancy, France; 6 R&D Unicancer, Paris, France

**Keywords:** Joint model, Informative dropout, Linear mixed model, Random intercept and slope model, Health-related quality of life, Longitudinal outcome, Clinical trials, Cancer

## Abstract

**Background:**

Patient-reported outcomes such as health-related quality of life (HRQoL) are increasingly used as endpoints in randomized cancer clinical trials. However, the patients often drop out so that observation of the HRQoL longitudinal outcome ends prematurely, leading to monotone missing data. The patients may drop out for various reasons including occurrence of toxicities, disease progression, or may die. In case of informative dropout, the usual linear mixed model analysis will produce biased estimates. Unbiased estimates cannot be obtained unless the dropout is jointly modeled with the longitudinal outcome, for instance by using a joint model composed of a linear mixed (sub)model linked to a survival (sub)model. Our objective was to investigate in a clinical trial context the consequences of using the most frequently used linear mixed model, the random intercept and slope model, rather than its corresponding joint model.

**Methods:**

We first illustrate and compare the models on data of patients with metastatic pancreatic cancer. We then perform a more formal comparison through a simulation study.

**Results:**

From the application, we derived hypotheses on the situations in which biases arise and on their nature. Through the simulation study, we confirmed and complemented these hypotheses and provided general explanations of the bias mechanisms.

**Conclusions:**

In particular, this article reveals how the linear mixed model fails in the typical situation where poor HRQoL is associated with an increased risk of dropout and the experimental treatment improves survival. Unlike the joint model, in this situation the linear mixed model will overestimate the HRQoL in both arms, but not equally, misestimating the difference between the HRQoL trajectories of the two arms to the disadvantage of the experimental arm.

**Supplementary Information:**

The online version contains supplementary material available at 10.1186/s12874-023-01846-3.

## Background

There is a growing interest in using patient-reported outcomes (PROs) in addition to objective endpoints when assessing the benefit of new treatments or new therapeutic strategies. In cancer clinical trials, health-related quality of life (HRQoL) has become an almost standard secondary endpoint and is even sometimes (in palliative or supportive care) a primary or co-primary endpoint. Usually, HRQoL is assessed by a self-administered questionnaire at different visit times during care and follow-up, and results in a longitudinal outcome analyzed using linear mixed models (LMMs).

However, assessment of HRQoL often ends prematurely at timepoints that differ between patients. The patients may simply stop completing the questionnaires, drop out for various reasons including occurrence of toxicities, disease progression, or die. In case of informative dropout, i.e., if the dropout is related to the HRQoL outcome, an LMM analysis will lead to biased estimates [1]. To avoid biases, the joint distribution of the HRQoL and dropout variables must be considered, for example through a joint model (JM) that consists of a linear mixed (sub)model for the longitudinal HRQoL outcome and a survival (sub)model for the time to dropout. By sharing parameters including the random effects, JMs allow for the association between a longitudinal outcome and a time to event [2]. They have been extensively used to make predictions on the occurrence of a clinical event while accounting for an endogenous time-dependent covariate (the longitudinal outcome), for example predicting prostate cancer recurrence using PSA (prostate-specific antigen) measurements [3]. In clinical trials, JMs are increasingly applied using a biomarker as the longitudinal outcome. In this context, they can provide more efficient estimates of the treatment effect on the longitudinal outcome and of the direct treatment effect on survival as well as less biased estimates of the overall treatment effect on survival [4]. In addition, JMs have been applied using a HRQoL longitudinal outcome and in particular have shown that part of the survival benefit of a treatment can be masked by the negative effect that the treatment has on patients’ HRQoL [5]. Nevertheless, applications of JMs where the primary interest is in the longitudinal outcome are less frequent; among them, those using HRQoL longitudinal outcomes in clinical trial settings are rare. Indeed, there is little knowledge of the impact of taking into account or not dropout on the estimation of the parameters that characterize the HRQoL outcome trajectory; when, how, and why will the usual LMM analysis produce misleading results? Will the biases be similar in the two treatment arms or could the results affect the between-arm comparison?

This article studies the practical implications of using an LMM to analyze a HRQoL longitudinal outcome in a randomized clinical trial where the patients may drop out.

In Section 2, we detail the models that will be compared: the random intercept and slope model, which is the most frequently used LMM in clinical trial settings, and its corresponding JM. Section 3 deals with PRODIGE 4/ACCORD 11, a randomized phase II-III clinical trial including patients with metastatic pancreatic cancer. We apply the two models to data from this trial and derive hypotheses on the situations in which using the LMM would lead to biased estimates of the HRQoL parameters. In Section 4, we conduct a simulation study considering different scenarios to validate (or invalidate) these hypotheses and more generally to study the bias mechanisms in depth. We discuss our findings in Section 5 and conclude in Section 6.

## Modeling the HRQoL longitudinal outcome

In the following, the longitudinal outcome to be considered consists of a HRQoL score and the corresponding variable is assumed to be continuous and normally distributed.

### Linear mixed model (LMM)

The usual approach to analyzing longitudinal HRQoL score data consists of using an LMM. According to its general form, the HRQoL score of patient *i* at time *t* is:

1 $$\begin{array}{rcll} Y_{i}\left(t\right)&=&\ \, {Y_{i}^{\star}}\left(t\right) & +\epsilon_{i}\left(t\right)\\ &=&\underset{\text{Mean trajectory}}{\underbrace{\beta^{\text{T}}X_{i}\left(t\right)}}+\;\underset{\text{Individual deviations}}{\underbrace{b_{i}^{\text{T}}Z_{i}\left(t\right)}} & +\epsilon_i\left(t\right)\end{array}$$

where ${Y}_i^{\star }(t)$ represents the true score value at time *t*, *β* and *b*_*i*_ are the vectors of the *p* fixed effects and *q* random effects, *X*_*i*_(*t*) and *Z*_*i*_(*t*) are the respective design vectors of size *p* and *q* containing the covariates at time *t*, and *ϵ*_*i*_(*t*) is the random error term at time *t*. It is assumed that *ϵ*_*i*_(*t*) ∼ *N*(0, *σ*^2^) and *b*_*i*_ ∼ *N*(0, *D*) where *D* is a *q* × *q* unstructured covariance matrix. Furthermore, the *ϵ*_*i*_(*t*) are mutually independent and independent of *b*_*i*_. Note that *Y*_*i*_(*t*) is only observed at time points *t*_*ij*_, *j* = 1, …, *n*_*i*_ where *n*_*i*_ is the number of HRQoL measurements for patient *i*, and that the *t*_*ij*_ and *n*_*i*_ can vary from a patient to another.

As it is commonly used in clinical trial settings, we have focused on the random coefficient model, or random intercept and slope model, which specifies the true HRQoL score trajectory as a linear function of time as follows:

2 $${Y}_i^{\star }(t)={\beta}_0+{\beta}_1t+{\beta}_2\left\{ ar{m}_i\times t\right\}+{b}_{0i}+{b}_{1i}t$$

where $$D=\left(\begin{array}{cc}{\sigma}_0^2& {\sigma}_{01}\\ {}{\sigma}_{01}& {\sigma}_1^2\end{array}\right)$$. The factor *arm*_*i*_ equals 1 if patient *i* belongs to the experimental arm receiving the new treatment and 0 if patient *i* belongs to the control arm receiving the standard treatment. The fixed intercept *β*_0_ represents the mean score at inclusion (*t* =0), the fixed slope *β*_1_ represents the score change by unit of time in the control arm, the interaction effect *β*_2_ represents the difference between the slopes of the experimental and control arms, and the random intercept *b*_0*i*_ and random slope *b*_1*i*_ represent individual deviations from the fixed intercept and fixed slope, respectively. Note that *β*_1_ + *β*_2_ corresponds to the slope in the experimental arm. Note also that there is no arm effect in the model because randomization normally ensures that baseline HRQoL is similar between the two arms.

### Joint model (JM)

To analyze longitudinal HRQoL score data taking into account the fact that observation ends with an event (dropout), an alternative approach consists of using a JM that links the LMM to a time-to-event model through shared parameters. In general, the latter is a proportional hazards model that includes the true current value of the longitudinal outcome as a covariate on the hazard function:

3 $${\lambda}_i(t)={\lambda}_0(t)\exp \left\{{\gamma}^T{W}_i+\alpha\ {Y}_i^{\star }(t)\right\}$$

where *λ*_0_(*t*) is the baseline hazard function, *W*_*i*_ is the vector of baseline time-independent covariates that includes the treatment arm and possibly other prognostic factors or covariates, *γ* is the vector of the corresponding effects, and *α* is the parameter that represents the association between the risk of event and the current true value of the longitudinal outcome ${Y}_i^{\star }(t)$.

We have considered the JM where the (sub)model for the longitudinal HRQoL outcome is given by Eqs. (1) and (2) and the survival (sub)model is given by:

4 $${\lambda}_i(t)={\lambda}_0(t)\exp \left\{{\gamma}_1\ {arm}_i+\alpha\ {Y}_i^{\star }(t)\right\}$$

with *γ*_1_ corresponding to the direct treatment effect on the risk of event. Note that due to the presence of ${Y}_i^{\star }(t)$, the quantities given by exp{*γ*_1_} and exp{*α*} are conditional hazard ratios (HRs) controlling for the random effects. The baseline hazard function was assumed to be piecewise constant (application of Section 3) or to follow a Weibull distribution (simulation study of Section 4).

### Predicted HRQoL score trajectories

From the estimated parameters of each model, one can obtain predicted values of the HRQoL score at any time *t*, thus one can depict the predicted HRQoL score trajectories. The predicted HRQoL score trajectory of patient *i* is given by:

5 $${\hat{Y}}_i(t)=\hat{\beta_0}+\hat{\beta_1}t+\hat{\beta_2}\left\{ ar{m}_i\times t\right\}+\hat{b_{0i}}+\hat{b_{1i}}t$$

However, the interest often lies on the predicted mean trajectories rather than on the predicted individual trajectories, that is on plotting on the same graph the mean trajectory in the experimental arm:

6 $${\hat{Y}}_{arm=1}(t)=\mathbb{E}\left(Y(t)| arm=1\right)=\hat{\beta_0}+\left(\hat{\beta_1}+\hat{\beta_2}\right)t$$

and the mean trajectory in the control arm:

7 $${\hat{Y}}_{arm=0}(t)=\mathbb{E}\left(Y(t)| arm=0\right)=\hat{\beta_0}+\hat{\beta_1}t$$

## The PRODIGE 4/ACCORD 11 clinical trial

### Description

PRODIGE4/ACCORD11 was a multicenter, randomized, phase II-III clinical trial comparing FOLFIRINOX (combination of folinic acid, fluorouracil, irinotecan, and oxaliplatin) and gemcitabine (reference regimen) as first-line chemotherapy for patients with metastatic pancreatic cancer. Detailed inclusion and exclusion criteria, as well as study design have been previously published [6]. Inclusion criteria included a measurable metastatic pancreatic adenocarcinoma, an Eastern Cooperative Oncologic Group (ECOG) performance status score of 0 or 1, and no prior chemotherapy. The primary endpoint for the phase III analysis was overall survival, and the secondary endpoints were progression-free survival, tumor response, safety, and HRQoL.

### HRQoL assessment

HRQoL was assessed by use of the European Organization for Research and Treatment of Cancer (EORTC) QLQ-C30 questionnaire (version 3.0) at inclusion, then every 2 weeks during treatment, then every 2 months until progression, then every 6 months until death or end of study. The QLQ-C30 is a 30-item self-administered cancer-specific questionnaire composed of five functional scales (physical, role, cognitive, emotional, and social functioning), nine symptom scales (fatigue, nausea and vomiting, pain, dyspnea, insomnia, appetite loss, constipation, diarrhea, financial difficulties) and a global health status/HRQoL scale [7]. The primary endpoint for the HRQoL analysis was global health status/HRQoL domain, and the secondary endpoints were physical, role, and social functioning, and fatigue and pain. For each scale, a standardized score ranging from 0 to 100 was calculated from the item responses as recommended by the EORTC [8]. A high score for the global health status/HRQoL and a functional scale represents respectively a high HRQoL and a high level of functioning and so is associated with a good HRQoL level; conversely, a high score for a symptom scale represents a high level of symptomatology and so is associated with a poor HRQoL level.

### Main findings

The main findings of the trial have been previously published [6]. All analyses were performed on the intention-to-treat (ITT) population that included 342 patients (*n* = 171 in the FOLFIRINOX experimental arm, *n* = 171 in the gemcitabine control arm). Demographic and baseline disease characteristics were similar in the two treatment arms, except for the number of measurable target lung metastases (fewer in the FOLFIRINOX arm than in the gemcitabine arm). Significant differences were found in overall survival (HR = 0.57, 95% confidence interval (CI): [0.45, 0.73], *p* < 0.001) and in progression-free survival (HR = 0.47, 95% CI: [0.37; 0.59], *p* < 0.001) in favor of the FOLFIRINOX arm. Median overall survival was 11.1 months (95% CI: [9.0; 13.1]) in the FOLFIRINOX arm and 6.8 months (95% CI: [5.5; 7.6]) in the gemcitabine arm. Median progression-free survival was 6.4 months (95% CI: [5.5; 7.2]) in the FOLFIRINOX arm versus 3.3 months (95% CI: [2.2; 3.6]) in the gemcitabine arm. However, more adverse events occurred in the FOLFIRINOX arm than in the gemcitabine arm. HRQoL in the two treatment arms was compared through a time-to-definitive-deterioration analysis with responder thresholds of 10 and 20 points to quantify an individual change [9]. A decreased risk of definitive deterioration in favor of the FOLFIRINOX arm was found in the six scales of interest: the global health status/HRQoL scale (HR = 2.3, *p* < 0.001), the physical (HR = 1.9, *p* =0.001), role (HR = 2.2, *p* < 0.001), and social (HR = 2.1, *p* < 0.001) functioning scales, and the fatigue (HR = 1.9, *p* =0.001) and pain (HR = 2.7, *p* < 0.001) symptom scales (values given for a 10-point threshold).

### Application of the LMM and JM to analyze longitudinal HRQoL data

We applied the LMM and the JM described in Section 2 to data from the PRODIGE 4/ACCORD 11 trial to analyze the HRQoL score evolution in the two treatment arms for each of the six scales of interest. Contrary to the LMM, the JM take into account the fact that death occurrence stopped the observation of the longitudinal HRQoL outcome. The analyses were performed on the 335 evaluable patients (FOLFIRINOX arm: *n* = 167, gemcitabine arm: *n* = 168) of the ITT population (i.e., with at least one HRQoL score measurement).

**Fig. 1:**
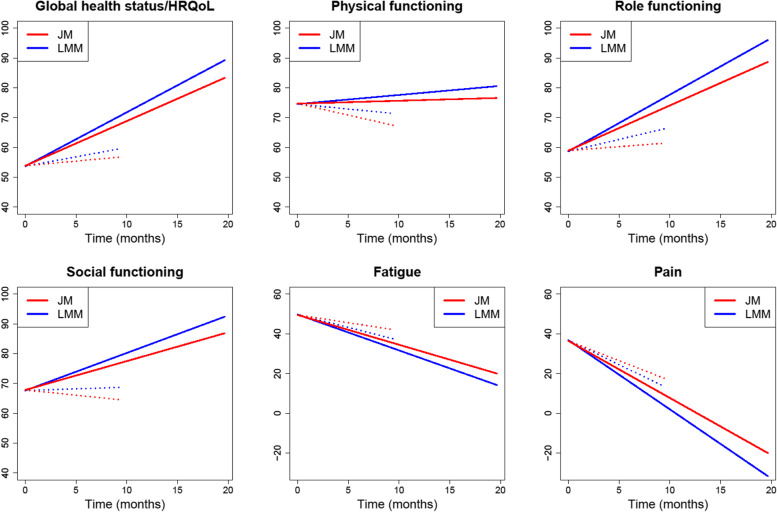
Predicted mean HRQoL score trajectories in the experimental (solid lines) and control (dotted lines) arms from the LMM and the JM fitting to the clinical trial PRODIGE 4/ACCORD 11 data for the six scales of interest. HRQoL, health-related quality of life; JM, joint model; LMM, linear mixed model

We used the R package nlme for the LMM (function lme) and the R package JM for the joint model (function jointModel with a piecewise-constant baseline hazard on seven intervals and a pseudo-adaptative Gauss–Hermite method with 15 quadrature points to approximate the integrals over the random effects). The main estimation results (*β*_1_, *β*_2_, *γ*_1_, and *α* parameters) are summarized in Table 1 and the predicted mean score trajectories are depicted in Fig. 1. The results concerning *β*_0_ and the variance parameters can be found in Supplementary Table 1.

**Table 1:** Main results of the LMM and JM fitted to the clinical trial data

	HRQoL score trajectory								Risk of dropout			
	Time effect				Arm-by-time interaction effect				Arm effect		Association with HRQoL	
	LMM		JM		LMM		JM		JM		JM	
	${\hat{\beta}}_1$ [95% CI]	*p*	${\hat{\beta}}_1$ [95% CI]	*p*	${\hat{\beta}}_2$ [95% CI]	*p*	${\hat{\beta}}_2$ [95% CI]	*p*	${\hat{\gamma}}_1$ [95% CI]	*p*	$\hat{\alpha}$ [95% CI]	*p*
GLOBAL HEALTH STATUS/HRQoL												
Global health status/HRQoL	0.63 [− 0.17; 1.42]	0.121	0.31 [− 0.45; 1.07]	0.420	1.18 [0.25; 2.10]	**0.013**	1.19 [0.34; 2.03]	**0.006**	−0.43 [− 0.72; − 0.15]	**0.003**	− 0.029 [− 0.041; − 0.016]	< **10**^−**4**^
FUNCTIONAL SCALES												
Physical functioning	− 0.33 [− 1.17; 0.51]	0.439	− 0.77 [− 1.68; 0.15]	0.101	0.64 [− 0.38; 1.66]	0.222	0.86 [− 0.12; 1.85]	0.086	− 0.66 [− 0.95; − 0.37]	< **10**^−**4**^	−0.028 [− 0.036; − 0.020]	< **10**^−**4**^
Role functioning	0.80 [− 0.38; 1.98]	0.183	0.27 [− 0.93; 1.46]	0.663	1.10 [− 0.27; 2.47]	0.116	1.25 [− 0.08; 2.57]	0.065	− 0.57 [− 0.86; − 0.29]	< **10**^−**4**^	− 0.018 [− 0.024; − 0.012]	< **10**^−**4**^
Social functioning	0.11 [− 0.92; 1.15]	0.831	− 0.35 [− 1.44; 0.75]	0.533	1.15 [− 0.07; 2.37]	0.065	1.32 [0.09; 2.55]	**0.036**	− 0.57 [− 0.84; − 0.29]	< **10**^−**4**^	−0.017 [− 0.022; − 0.012]	< **10**^−**4**^
SYMPTOM SCALES												
Fatigue	− 1.30 [− 2.35; − 0.24]	**0.016**	−0.78 [− 1.84; 0.28]	0.148	−0.51 [− 1.76; 0.74]	0.422	− 0.72 [− 1.90; 0.47]	0.235	−0.63 [− 0.91; − 0.35]	< **10**^−**4**^	0.019 [0.012; 0.026]	< **10**^−**4**^
Pain	−2.47 [−3.54; − 1.40]	**< 10**^−**3**^	−1.99 [− 3.01; − 0.98]	< **10**^−**3**^	−1.01 [− 2.21; 0.19]	0.098	−0.88 [− 2.00; 0.24]	0.125	− 0.50 [− 0.77; − 0.22]	< **10**^−**3**^	0.017 [0.007; 0.026]	< **10**^−**3**^

*HRQoL* Health-related quality of life, *JM* Joint model, *LMM* Linear mixed model, *CI* Confidence interval

### Clinical comments

For all scales except physical functioning, both models found a tendency toward improvement in the HRQoL in the control arm – that is, a score increase (${\hat{\beta}}_1$ > 0) in the global health status/HRQoL and the functional scales and a score decrease (${\hat{\beta}}_1$ < 0) in the symptom scales. Both models detected a significant improvement of pain symptoms (*p* < 10^−3^) but only the LMM found a significant improvement of fatigue symptoms (*p* = 0.016).

Both models found that all dimensions of HRQoL were improved in the experimental arm versus the control arm (i.e., ${\hat{\beta}}_2$ > 0 for the global health status/HRQoL and the functional scales and ${\hat{\beta}}_2$ < 0 for the symptom scales). Both models detected that this arm-by-time effect was significant for the global health status/HRQoL (*p* = 0.013 for the LMM and *p* = 0.006 for the JM); only the JM detected a significant effect for the social functioning scale (*p* = 0.036).

For all the HRQoL dimensions, the JM detected a significant and protective (i.e., ${\hat{\gamma}}_1$ < 0) direct effect of the experimental treatment on the risk of death. We found that risk of death adjusted for the current HRQoL score was multiplied by a HR going from exp(−0.43) = 0.65 for global health status/HRQoL to exp(− 0.66) = 0.52 for physical functioning. This is consistent with the primary endpoint analysis that had found a marginal risk of death multiplied by a HR of 0.57 in the experimental arm compared with the control arm (see Section 3.3).

For all the HRQoL dimensions, the JM found that a poorer level was significantly associated with an increased risk of death. This negative association was strongest for the global health status/HRQoL scale, with an estimated value of $\hat{\alpha}$ = − 0.029, (*p* < 10^−4^), meaning that a diminution of 8.33 points, which is the difference between two adjacent possible score values in this scale, corresponds to a risk increase of exp(0.029 × 8.33) = 1.27. A consequence of this association can be observed in Fig. 1. In the control arm (dotted lines), where HRQoL over time is poorer than in the experimental arm (solid lines), the predicted mean trajectories go up to approximately 10 months (vs. 20 months in the experimental arm). Indeed, the time interval during which there are available HRQoL score data is shorter in the control arm than in the experimental arm due to earlier deaths.

### Methodological comments

Compared with the LMM, the JM found that HRQoL in the control arm improved less (global health status/HRQoL: ${\hat{\beta}}_1$ = 0.31 vs. 0.63; role functioning: ${\hat{\beta}}_1$ = 0.27 vs. 0.80; fatigue: ${\hat{\beta}}_1$ = − 0.78 vs. −1.30; pain: ${\hat{\beta}}_1$ = − 1.99 vs. −2.47), deteriorated more (physical functioning: ${\hat{\beta}}_1$ = − 0.77 vs. −0.33) or deteriorated instead of improved (social functioning: ${\hat{\beta}}_1$ = − 0.35 vs. 0.11). These results suggest that the LMM would overestimate the slope parameter *β*_1_ in the global health status/HRQoL and functional scales and would underestimate it in the symptom scales. It is important to note that fatigue decreased non-significantly according to the JM while the (presumed) underestimation of the LMM made ${\hat{\beta}}_1$ cross the level of significance.

Compared with the LMM, for five out of six scales (all except for pain) the JM found a larger difference (in favor of the experimental treatment) of the HRQoL score trajectory between arms and smaller associated *p*-values. This result suggests that the LMM would underestimate the arm-by-time interaction parameter *β*_2_. In addition, the larger the direct arm effect *γ*_1_ was on the risk of death, the larger the difference was between the *β*_2_ estimates of the LMM and the JM. It should be noted that a consequence of the (presumed) underestimation of the arm-by-time interaction effect *β*_2_ by the LMM is that this model found this effect to be non-significant in the social functioning scale, whereas the JM found this effect to be significant.

From the previous comments, we can derive the following hypotheses:

- If there is a negative association between HRQoL and the risk of event (dropout), then the LMM will overestimate (global health status/HRQoL and functional scales) or underestimate (symptomatic scales) the time effect *β*_1_, leading to an overly optimistic predicted HRQoL score trajectory in the control arm.
- If there is also a protective effect *γ*_1_ of the new treatment on the risk of event (dropout), then the LMM will underestimate (global health status/HRQoL and functional scales) or overestimate (symptomatic scales) the arm-by-time interaction effect *β*_2_. In particular, where the HRQoL level over time is better in the experimental arm compared with the control arm, the LMM will diminish the beneficial treatment effect on HRQoL.

A simulation study is needed to validate these statements; this is the purpose of the next section.

## Simulation study

We conducted a simulation study to compare both models under several scenarios. We based the design of the simulations on PRODIGE 4/ACCORD 11 and our knowledge about various clinical trials. For each scenario, we generated 1000 datasets of 500 patients randomly assigned to two treatment arms with 250 patients per arm. To compare the models, we calculated for each estimated parameter $\hat{\beta}$ the following criteria: the mean value $\overline{\hat{\beta}}$, the bias $\overline{\hat{\beta}}-\beta$, the relative bias $\left(\overline{\hat{\beta}}-\beta \right)/\beta$, the root mean square error (RMSE) $\sqrt{\overline{{\left(\hat{\beta}-\beta \right)}^2}}$, and the coverage rate, i.e., the proportion of samples for which the 95% CI includes the true value *β*.

### Design

#### Visit times

For all patients, there were 15 scheduled visit times for HRQoL assessment at: inclusion, month 1, month 2, month 3, month 6, month 9, month 12, month 18, and then every 6 months until administrative censoring (end of study). For each patient *i*, we generated the individual visit times *t*_*ij*_ where *j* is the visit number (*j* = 1, …, 15). At inclusion, *t*_*i*1_ = 0 ∀ *i*, and, for *j* > 1, the *t*_*ij*_ were uniformly distributed: within ±3 days of scheduled visits in months 1 to 3, ±7 days of scheduled visits in months 6 to 12, and ± 14 days of scheduled visits at later timepoints.

#### Longitudinal HRQoL outcome and time to dropout

For each patient *i*, we generated the longitudinal HRQoL outcome following the JM defined by Eqs. (1) and (2) at the visit times *t*_*ij*_, *j* = 1, …, 15. We then generated the dropout time from Eq. (4) using a Weibull distribution for the baseline hazard; that is, with *λ*_0_(*t*) = *ϕt*^*ϕ* − 1^ exp(*γ*_0_) where *ϕ* was the shape parameter and exp(*γ*_0_) corresponded to the scale parameter (we used an exponential parametrization for the scale parameter to be consistent with the JM package that estimates *γ*_0_ using an intercept in the exponentiated term of the hazard function). As usual inverse transform sampling was not straightforward, in particular because of the time-varying covariate *Y*^⋆^(*t*), we used a method implemented in the R package simsurv that allows survival times to be generated from complex models such as JMs [10].

If dropout occurred before administrative censoring, we removed all the HRQoL data for visit times after the time to dropout.

#### Parameter values

The choice of the parameter values was based on the estimates from the JM applied to the PRODIGE 4/ACCORD 11 trial data for the global health status/HRQoL scale while assuming a Weibull distribution to specify the baseline hazard function (main results depicted in Supplementary Table 2). In Scenario 0, all the parameter values were set to the estimates from the application rounded to the second decimal place; in Scenarios 1, 2, 3, 4, and 5, some parameter values deviated from the application (as detailed in the next section).

### Scenarios

#### General principle

We have considered five different scenarios. The “PRODIGE 4/ACCORD 11”-like scenario (Scenario 0) aimed to verify that the simulation results were consistent with the application results of Section 3.4. The other scenarios (1, 2, 3, 4, and 5) aimed to confirm and generalize the statements derived from the methodological comparison of Section 3.6.

#### HRQoL trajectory parameters

**Fig. 2:**
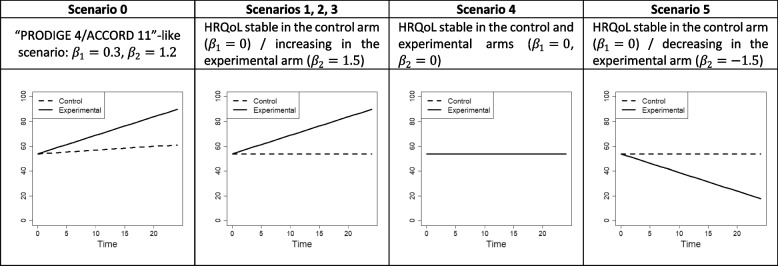
Mean trajectories of the true HRQoL score value in both arms considered in the simulation study (i.e., representation of $\mathbb{E}\left(\left({Y}^{\star}\Big(t\right)\right)={\beta}_0+{\beta}_1t+{\beta}_2\left\{ arm\times t\right\}$ according to *t* with *t* the time in months and *arm* the treatment arm indicator). HRQoL, health-related quality of life

In all scenarios, the variance parameters were set to the rounded estimates from PRODIGE 4/ACCORD 11: *σ*_0_ = 15.2, *σ*_1_ = 2.1, *ρ*_01_ = − 0.4 (with *ρ*_01_ = *σ*_01_/*σ*_0_*σ*_1_), *σ* = 13.5. In Scenario 0, all other parameters were also set to the rounded estimates from this application: *β*_0_ = 53.9, *β*_1_ = 0.3, and *β*_2_ = 1.2, corresponding to a HRQoL value that increased slightly over time in the control arm and substantially in the experimental arm. In the other scenarios, the mean HRQoL value at baseline was *β*_0_ = 50, and then HRQoL values were constant in the control arm (*β*_1_ = 0) and increased (*β*_2_ = 1.5 in Scenarios 1, 2, 3), were constant (*β*_2_ = 0 in Scenario 4), or decreased (*β*_2_ = − 1.5 in Scenario 5) in the experimental arm. The mean trajectory of the HRQoL value in each treatment arm is depicted in Fig. 2 for all scenarios.

#### Risk-of-dropout parameters

In Scenario 0, the regression parameters were set to the rounded estimates from PRODIGE 4/ACCORD 11. The arm effect was *γ*_1_ = − 0.4, corresponding with a reduced risk of dropout due to the direct effect of the experimental treatment, and the association parameter was *α* = − 0.02, meaning that an increase in the HRQoL value was associated with a reduced risk of dropout. In Scenario 1, risk of dropout was the same in both treatment arms (*γ*_1_ = 0) and there was no association between HRQoL and dropout (*α* = 0). In Scenario 2, the risk of dropout was still the same in both treatment arms (*γ*_1_ = 0) but the current value of HRQoL was negatively associated with the risk of dropout (*α* = − 0.03). In Scenarios 3, 4, and 5, HRQoL and dropout were still negatively associated (*α* = − 0.03) and, in addition, the experimental treatment reduced the risk of dropout (*γ*_1_ = − 0.7).

The Weibull parameters for the baseline hazard function were set to *ϕ* = 1.6 and *γ*_0_ = − 2.2 in Scenarios 0, 2, 3, 4, and 5 based on the PRODIGE 4/ACCORD 11 application. We replaced them with *ϕ* = 1.7 and *γ*_0_ = − 4.1 in Scenario 1, where there was no association between HRQoL and dropout (*α* = 0), in order to obtain an overall hazard function comparable to those of the other scenarios.

**Fig. 3:**
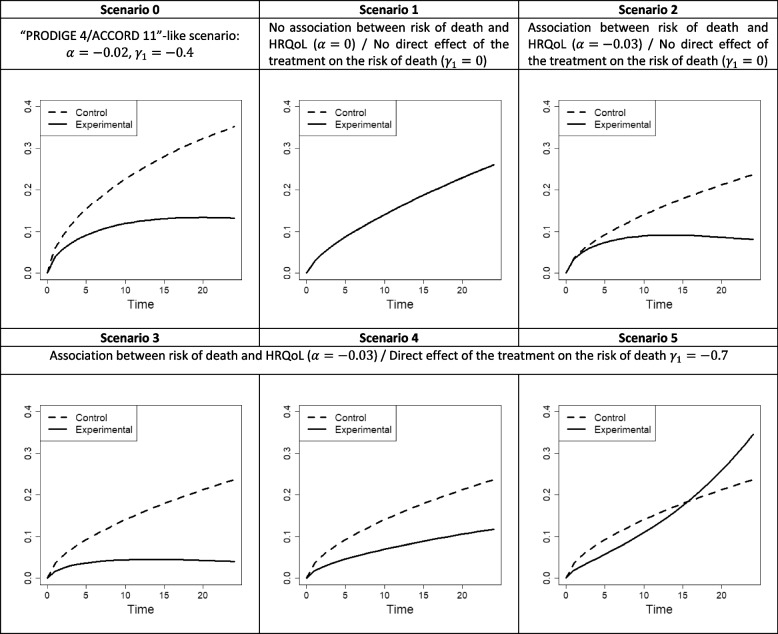
Representation of the hazard functions considered in the simulation study for both arms where the current HRQoL true value is set to its theoretical mean, i.e., $\lambda \left(t| arm,\mathbb{E}\left(\left({Y}^{\star}\Big(t\right)\right)\right)={\lambda}_0(t)\exp \left\{{\gamma}_1\ arm+\alpha\ \left({\beta}_0+{\beta}_1t+{\beta}_2\left\{ arm\times t\right\}\right)\right\}$ where *λ*_0_(*t*) = *ϕt*^*ϕ* − 1^ exp(*γ*_0_) with *ϕ* and exp(*γ*_0_) the shape and scale parameters, respectively, according to *t* with *t* the time in months and *arm* the treatment arm indicator. HRQoL, health-related quality of life

Figure 3 depicts for each scenario the hazard function in both treatment arms with a current HRQoL true value set to its theoretical mean – that is, the hazard function given by: *λ*_0_(*t*) exp {*γ*_1_*arm* + *α*(*β*_0_ + *β*_1_*t* + *β*_2_{*arm* × *t*})}. Note that the curves are not the same in Scenarios 3, 4, and 5, while all the parameters governing the risk of dropout are the same. This comes from different values of the HRQoL covariate since, as can be seen in Fig. 2, the HRQoL trajectory in the experimental arm varies: it increases, is constant, and decreases in Scenarios 3, 4, and 5, respectively. Depending on the scenario, the mean of the median survival times in the 1000 simulations varied from 6.2 to 9 months for the control arm and from 8.7 to 17 months for the experimental arm (see Supplementary Table 3).

### Results

**Fig. 4:**
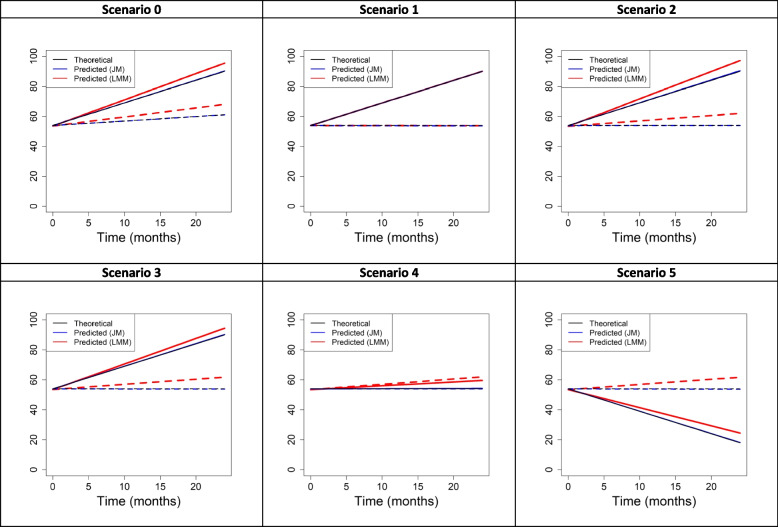
HRQoL theoretical mean trajectories (in black) and mean of the HRQoL predicted mean trajectories over the simulations (in color) in the control arm (dashed lines) and the experimental arm (solid lines), i.e., representation of the functions given by, respectively, *β*_0_ + *β*_1_*t* + *β*_2_{*arm* × *t*} and $\overline{{\hat{\beta}}_0}+\overline{{\hat{\beta}}_1}t+\overline{{\hat{\beta}}_2}\left\{ arm\times t\right\}$, according to *t* with *t* the time in months and *arm* the treatment arm indicator. HRQoL, health-related quality of life; JM, joint model; LMM, linear mixed model

The results (mean, bias, relative bias, RMSE, and coverage rate) concerning the main parameters, *β*_1_, *β*_2_, *γ*_1_, and *α*, are summarized in Table 2; for readability, the table also details the results (mean, bias, and relative bias) for *β*_1_ + *β*_2_. Supplementary Table 4 details the results concerning the other parameters (intercept, variance parameters, and Weibull parameters). Figure 4 depicts the mean of the predicted mean HRQoL trajectories from the two models by treatment arm.

**Table 2:** Results^a^ of the simulation study on the main parameters^b^

		LMM					JM				
	True	Mean	Bias	RB	RMSE	Cov.	Mean	Bias	RB	RMSE	Cov.
**SCENARIO 0**											
**HRQoL**											
*β*_1_	0.3	0.603	0.303	1.010	0.371	70.8	0.297	− 0.003	− 0.011	0.22	95.7
*β*_2_	1.2	1.141	−0.059	− 0.049	0.287	93.1	1.215	0.015	0.012	0.284	93.3
*β*_1_ + *β*_2_	1.5	1.744	0.244	0.163	–	–	1.512	0.012	0.008	–	–
**Risk of dropout**											
*γ*_1_	− 0.4	–	–	–	–	–	− 0.403	− 0.003	0.007	0.103	93.8
*α*	−0.02	–	–	–	–	–	− 0.02	0	0.003	0.003	95.2
**SCENARIO 1**											
**HRQoL**											
*β*_1_	0	−0.007	−0.007	NA	0.183	94.1	−0.008	−0.008	NA	0.184	94.2
*β*_2_	1.5	1.508	0.008	0.005	0.253	93.4	1.508	0.008	0.005	0.253	93.3
*β*_1_ + *β*_2_	1.5	1.501	0.001	0.001	–	–	1.5	0	0	–	–
**Risk of dropout**											
*γ*_1_	0	–	–	–	–	–	−0.002	−0.002	NA	0.102	93.4
*α*	0	–	–	–	–	–	0	0	NA	0.002	94.9
**SCENARIO 2**											
**HRQoL**											
*β*_1_	0	0.356	0.356	NA	0.404	52.7	0.004	0.004	NA	0.198	94.3
*β*_2_	1.5	1.465	−0.035	−0.023	0.255	93.1	1.517	0.017	0.011	0.260	93.4
*β*_1_ + *β*_2_	1.5	1.821	0.321	0.214	–	–	1.521	0.021	0.014	–	–
**Risk of dropout**											
*γ*_1_	0	–	–	–	–	–	−0.003	− 0.003	NA	0.110	94.3
*α*	−0.03	–	–	–	–	–	−0.03	0	0	0.003	95.5
**SCENARIO 3**											
**HRQoL**											
*β*_1_	0	0.343	0.343	NA	0.391	53.0	−0.003	−0.003	NA	0.193	94.2
*β*_2_	1.5	1.359	−0.141	− 0.094	0.274	88.6	1.515	0.015	0.01	0.240	93.0
*β*_1_ + *β*_2_	1.5	1.702	0.202	0.135	–	–	1.512	0.012	0.008	–	–
**Risk of dropout**											
*γ*_1_	−0.7	–	–	–	–	–	− 0.704	−0.004	0.006	0.115	94.4
*α*	−0.03	–	–	–	–	–	−0.03	0	0.001	0.003	95.4
**SCENARIO 4**											
**HRQoL**											
*β*_1_	0	0.352	0.352	NA	0.397	51.8	0.002	0.002	NA	0.190	95.2
*β*_2_	0	−0.098	−0.098	NA	0.255	91.7	0.014	0.014	NA	0.241	93.8
*β*_1_ + *β*_2_	0	0.254	0.254	NA	–	–	0.016	0.016	NA	–	–
**Risk of dropout**											
*γ*_1_	−0.7	–	–	–	–	–	− 0.706	−0.006	0.009	0.108	94.1
*α*	−0.03	–	–	–	–	–	−0.030	0.000	0.002	0.003	94.8
**SCENARIO 5**											
**HRQoL**											
*β*_1_	0	0.342	0.342	NA	0.39	54.2	−0.009	−0.009	NA	0.194	94.8
*β*_2_	−1.5	−1.551	−0.051	0.034	0.245	93.9	−1.482	0.018	−0.012	0.245	94.4
*β*_1_ + *β*_2_	−1.5	−1.209	0.291	0.582	–	–	−1.491	0.009	−0.006	–	–
**Risk of dropout**											
*γ*_1_	−0.7	–	–	–	–	–	−0.7	0	0	0.115	93.9
*α*	−0.03	–	–	–	–	–	−0.03	0	−0.003	0.003	94.5

^a^mean, bias, relative bias (RB), root mean square error (RMSE) and coverage rate (Cov.) based on 1000 generated datasets of 500 patients

^b^ *β*_1_ and *β*_2_: time and interaction parameters characterizing the slope of the HRQoL trajectory in the control and experimental arms; *γ*_1_ and *α*: effects of treatment and HRQoL on risk of dropout

*HRQoL,* Health-related quality of life; *JM,* Joint model; *LMM,* Linear mixed model

As can be seen in Table 2, the JM provided unbiased estimates of the main HRQoL parameters in all scenarios, in contrast with the LMM (which provided biased estimates in all scenarios other than Scenario 1). The bias and RMSE from the LMM were in general also larger than those from the JM for the other HRQoL parameters, though to a lesser extent than for *β*_1_ and *β*_2_ (see Supplementary Table 4). The JM also estimated the effects of HRQoL and treatment on the risk of dropout, *α* and *γ*_1_, as well as the baseline hazard parameters, *γ*_0_ and *ϕ,* with almost zero biases and coverage rates close to 95% for all scenarios.

In-depth comments of the results scenario by scenario are given below.

#### Scenario 0

In the “PRODIGE 4/ACCORD 11”-like scenario, HRQoL increased slightly over time in the control arm (slope: *β*_1_ = 0.3) and substantially in the experimental arm (slope: *β*_1_ + *β*_2_ = 1.5). The new treatment had a protective effect on the risk of dropout (*γ*_1_ = − 0.4), and an increase in the current value of HRQoL was associated with a reduced risk of dropout (*α* = − 0.02). The results were consistent with those found in the global health status/HRQoL scale in the application in Section 3: the LMM overestimated the slope parameter *β*_1_ = 0.3 ($\overline{{\hat{\beta}}_1}$ = 0.603 compared with $\overline{{\hat{\beta}}_1}$ = 0.297 with the JM) and underestimated the interaction parameter *β*_2_ = 1.2 ($\overline{{\hat{\beta}}_2}$ = 1.141 compared with $\overline{{\hat{\beta}}_2}$ = 1.215 with the JM). Consequently, the LMM overestimated the slope in the experimental arm (bias for *β*_1_ + *β*_2_: 0.244) but to a lesser extent than in the control arm (bias for *β*_1_: 0.303). The *β*_1_ coverage rate of the LMM deviated from the nominal level of 95%, unlike the JM (70.8% vs. 95.7%).

#### Scenario 1

Scenario 1 was the scenario of reference for subsequent Scenarios 2, 3, 4, and 5. In all these five scenarios, HRQoL in the control arm was constant over time and HRQoL in the experimental arm increased (Scenarios 1, 2, 3), was constant (Scenario 4), or decreased (Scenario 5). HRQoL was independent of risk of dropout (i.e., dropout was non-informative) in Scenario 1 (*α*=0), in contrast to Scenarios 2, 3, 4, and 5, so there was no need to use the JM rather than using its two linear mixed and survival submodels separately. In this scenario, the JM performed roughly as well as the LMM regarding the HRQoL parameters. The RMSEs were 0.184 (JM) and 0.183 (LMM) for *β*_1_, and 0.253 (both models) for *β*_2_. The JM also performed well regarding the risk-of-dropout parameters, with almost zero biases for *γ*_1_ and *α*.

#### Scenario 2

Unlike in Scenario 1, in Scenario 2 there was a negative association between the current value of HRQoL and risk of dropout (*α* = − 0.03). This led to an LMM mean overestimation of 0.356 for the HRQoL parameter *β*_1_ (=0), while the bias from the JM estimate was almost zero (0.004). The *β*_1_ coverage rate using the LMM was far from 95% (52.7% vs. 94.3% for the JM). For the HRQoL parameter *β*_2_, the relative bias from the LMM was doubled (in absolute value) compared with that from the JM (− 0.023 vs. 0.011). However, the absolute bias from the LMM was not substantial (− 0.035). This slight underestimation of *β*_2_ resulted in a slope overestimation that was slightly less important in the experimental arm (bias of *β*_1_ + *β*_2_: 0.321) than in the control arm (bias of *β*_1_: 0.356). It is noticeable that the slope overestimation is less important where the risk of dropout is lower; that is, in the experimental arm (see Fig. 3). In fact, even though the new treatment had no direct effect on the risk of dropout (*γ*_1_ = 0), it had a protective indirect effect through higher values of HRQoL in the experimental arm than in the control arm (see Fig. 2).

#### Scenario 3

In Scenario 3, the LMM behaved as it did in Scenario 2 as regards the *β*_1_ parameter, giving a bias of 0.343. Indeed, the HRQoL trajectory in the control arm was the same in the two scenarios, as was the association effect between the current value of HRQoL and risk of dropout (*α* = − 0.03). In contrast with Scenario 2, in Scenario 3 the new treatment had a protective effect on the risk of dropout (*γ*_1_ = − 0.7). Consequently, the LMM underestimated the interaction parameter *β*_2_ by 0.141 on average, with a corresponding relative bias of − 0.094 (vs. 0.010 using the JM) and a coverage rate of 88.6% (vs. 93% using the JM). Notice that, in comparison with Scenario 2, a similar overestimation of the slope in the control arm *β*_1_ associated with a larger underestimation of *β*_2_ led to a smaller overestimation of the slope governing the HRQoL trajectory in the experimental arm (bias of *β*_1_ + *β*_2_: 0.202). The lower bias of the slope *β*_1_ + *β*_2_ can be related to the decreased risk of dropout that is present in the experimental arm due to the additional treatment effect (see Fig. 3).

#### Scenarios 4 and 5

As expected, the LMM behaved in Scenarios 4 and 5 as it did in Scenario 3 (and 2) as regards the *β*_1_ parameter, since neither the HRQoL trajectory in the control arm nor the association between the current HRQoL value and risk of dropout had changed. As in Scenario 3, the new treatment was protective on the risk of dropout (*γ*_1_ = − 0.7 in Scenarios 3, 4, and 5) but, instead of benefiting HRQoL (*β*_2_ = 1.5 in Scenario 3), it had no effect in Scenario 4 (*β*_2_ = 0) and was deleterious in Scenario 5 (*β*_2_ = − 1.5). This led to ascending sizes of bias for the slope *β*_1_ + *β*_2_ in Scenarios 3, 4, and 5 (0.202, 0.254, and 0.291, respectively), which can be related to the increased risk of dropout in the experimental arm due to the indirect treatment effect through the HRQoL values. Conversely, sizes of bias for *β*_2_ descended in the three scenarios (− 0.141, − 0.098, and − 0.051, respectively).

#### Summary and further comments

The results of Scenario 0 confirmed that in the application to the PRODIGE 4/ACCORD 11 data, the LMM would have overestimated the slope parameter *β*_1_ and underestimated the interaction parameter *β*_2_ in the global health status/HRQoL scale. More generally, the results of the subsequent scenarios were consistent with the hypotheses derived from the application. In the reference Scenario 1 where there was no association between HRQoL and risk of dropout, the LMM performed well (as did the JM). In Scenarios 2, 3, 4, and 5, where a low current HRQoL value was associated with an increased risk of dropout, the LMM produced biased results for the HRQoL parameters *β*_1_ and *β*_2_, which the JM did not. In fact, to understand the bias of *β*_2_, it should be seen as the difference between the biases of the *β*_1_ and the *β*_1_ + *β*_2_ slopes governing the HRQoL trajectories in the control and experimental arms, respectively.

Scenarios 2, 3, 4, and 5 have shown that for a given degree of association (namely, *α* = − 0.03), the size of the LMM bias increases with the risk of dropout. Hence, the size of the bias varies with the factors affecting the risk of dropout, of which there were two in our simulation study: *γ*_1_, the direct effect of the treatment, and ${Y}_i^{\star }(t)$, the current HRQoL value. Scenarios 2 and 3 were similar except in the case of the treatment effect *γ*_1_: this was null in Scenario 2 but protective in Scenario 3. This implied a lower risk of dropout in the experimental arm in Scenario 3 and, consequently, a lower bias of the slope *β*_1_ + *β*_2_. Similarly, in Scenario 4, nothing differentiated the two arms (which had the same constant HRQoL trajectories) apart from the effect of the treatment on dropout, *γ*_1_, which was protective. This implied that the slope governing the HRQoL trajectory was less biased in the experimental arm (bias of *β*_1_ + *β*_2_: 0.254) than in the control arm (bias of *β*_1_: 0.352). Secondly, the size of the bias varied with the HRQoL itself, since ${Y}_i^{\star }(t)$ also impacted the risk of dropout. This is obvious in Scenarios 3, 4, and 5; these scenarios exhibited different HRQoL trajectories in the experimental arm but the same direct effect of the treatment on the risk of dropout. In these scenarios, the HRQoL trajectory in the experimental arm was increasing, constant, and decreasing, respectively, leading to ascending sizes of bias for the slope governing the HRQoL trajectory in this arm, *β*_1_ + *β*_2_. Similarly, in Scenario 2 where the treatment had no direct effect on the risk of dropout, the risk of dropout was lower in the experimental arm than in the control arm because of a higher HRQoL trajectory. This led to a slope that was less biased in the experimental arm (bias for *β*_1_ + *β*_2_: 0.321) than in the control arm (bias for *β*_1_: 0.356). Finally, it can be noted that in Scenarios 2, 3, 4, and 5, where the risk of dropout in the control arm was the same (since the HRQoL score trajectories were the same and, of course, there was no treatment effect), the biases for *β*_1_, the slope governing the HRQoL trajectory in the control arm, were similar (0.356, 0.343, 0.352, and 0.342, respectively).

## Discussion

This article focuses on the analysis of longitudinal HRQoL data in cancer clinical trials where observation may be interrupted during treatment or follow-up. We have shown that as soon as the risk of dropout is associated with HRQoL, the LMM typically used for data analysis will produce biased estimates. Obtaining unbiased results cannot be achieved without jointly modeling the dropout and the HRQoL longitudinal outcome.

We first compared the LMM with a JM on HRQoL data of patients with metastatic pancreatic cancer who may drop out before end of study due to death. The JM found a significant association between HRQoL and survival for the six analyzed scales. Moreover, the two models differed in the estimation of HRQoL parameters so that the main parameters of interest, *β*_1_ and *β*_2_, could be significant using the JM but non-significant using the LMM (social functioning), and vice versa (fatigue). A limitation of this application stems from the fact that death is not an ordinary dropout since the HRQoL outcome is not unobserved but cannot exist after death. Contrary to the LMM, the JM account for the dependence between the longitudinal outcome and the dropout but both models implicitly impute HRQoL data beyond dropout. Actually, if a longitudinal outcome is truncated by death rather than ordinary dropout and if the primary interest is in the longitudinal outcome rather than in the survival outcome, other kinds of models could be more relevant than JMs. In particular, the RCA (regression conditioning of being alive) models produce estimates regarding the surviving population [11, 12] but, at our knowledge, they have not been implemented in standard software. Another alternative would be stratifying the analysis by the survival time using pattern mixture models so that mean HRQoL trajectories could be plotted by groups defined by the time of death [13]. It should be noted that, because they use future survival information, pattern mixture models can be used for description but not to predict trajectories. Note that pattern mixture models are also an alternative to take into account ordinary dropout as well as selection models, but we found more advantages in using joint models [14].

In spite of the limitation we have just mentioned, the results of our application made it possible to derive two hypotheses on the bias mechanisms arising in presence of dropout, which we then confirmed and complemented by use of a simulation study.

The first hypothesis was that a negative association between HRQoL and risk of dropout would result in an overestimation of *β*_1_, the slope governing the HRQoL score trajectory in the control arm. We confirmed this in the simulation study and deduced that the same assertion holds for *β*_1_ + *β*_2_, the slope governing the HRQoL score trajectory in the experimental arm. Dropout is informative: the patients with the lowest levels of HRQoL are the most likely to drop out early, so will contribute only weakly to the likelihood function. The second hypothesis was that a protective effect of the new treatment on the risk of dropout would result in an underestimation of *β*_2_, the arm-by-time interaction effect (slope difference between experimental and control arms). The simulation study confirmed this and also revealed that to understand why and how *β*_2_ is biased, the bias of the sum of *β*_1_ + *β*_2_, rather than the bias of *β*_1_, should be the focus. No bias for *β*_2_ means that the slopes in both arms are equally overestimated, maintaining the correct difference between the slopes. Accordingly, the underestimation of *β*_2_ observed in the application would be the result of a lower slope overestimation in the experimental arm than in the control arm, due to a longer time before dropout.

Indeed, in addition to the degree of association between the current value of HRQoL and risk of dropout (*α* parameter), the simulation study revealed that the size of the biases in the HRQoL slope estimates increases with the risk of dropout. Thus, the size of the bias depends on the factors affecting the risk of dropout; the first is the direct effect of the treatment on the risk of dropout, *γ*_1_. If the treatment has a protective effect, the risk of dropout will be lower in the experimental arm than in the control arm, implying that the bias will be smaller for *β*_1_ + *β*_2_ than for *β*_1_. The second factor affecting the risk of dropout is the longitudinal HRQoL outcome itself, through the variable ${Y}_i^{\star }(t)$. Consequently, if the HRQoL score trajectories are different for the two arms, the slope bias will be largest for the arm with the poorest HRQoL values. HRQoL participates here in an indirect effect of the treatment on the risk of dropout. In practice, the direct and indirect treatment effects act together, and how the slope will be biased in one arm compared with the other will in fact depend on the overall treatment effect on the risk of dropout.

In a JM, the overall treatment effect corresponds to (the logarithm of) a time-varying HR between the experimental and control arms. For two patients sharing the same random effects, the overall treatment effect in the considered JM reduces to *γ*_1_ + *α β*_2_*t*, which corresponds to the sum of the direct and indirect treatment effects. This HR has a subject-specific interpretation since the model controls for the random effects when calculating it. In other applications where the focus is on the survival submodel – typically, an application where a longitudinal biomarker serves to predict progression or death – an estimate of an overall treatment effect with a marginal interpretation could be preferred. To achieve this, van Oudenhoven et al. [15] recently proposed a method that is implemented in the JM package. However, the reason for using a JM in the present work was to obtain fair estimates of the HRQoL parameters, so we mainly focused on the linear mixed submodel.

Our findings could be extended to other JMs in many aspects. For example, the survival submodel could be adjusted on additional prognostic factors, and the linear mixed submodel could include other variables such as the treatment arm. In the latter case, the indirect treatment effect on the risk of dropout would take the form *α*(*β*_3_ + *β*_2_*t*) where *β*_3_ is the baseline arm effect. Our conclusions are based on a HRQoL score for which high values are associated with a high level of HRQoL and low values are associated with a poor level of HRQoL, such as the scores from the global health status/HRQoL scale or the functional scales of the QLQ-C30. The same conclusions hold in the reverse situation of a scale in which high values are associated with a poor level of HRQoL and low values are associated with a high level of HRQoL, such as the score from the symptomatic scales of the QLQ-C30. In this case, the association parameter *α* would be positive instead of negative when a poor level of HRQoL is associated with an increased risk of dropout. Obviously, the present works remains valid for PROs other than HRQoL.

Finally, note that we focused on a LMM and a JM assuming a linear relationship between the HRQoL outcome and time (random coefficient model). Nevertheless, we encourage the use of models allowing for a flexible trajectory of the HRQoL outcome (for example, based on splines). Indeed, an analysis assuming a simplistic functional form for the HRQoL outcome could miss information and provide misleading results [16]. Note that the bias mechanisms that have been revealed in this article should operate similarly in case of flexible models; although the parameters would lose their interpretability, our findings could probably by extended to the resulting predicted trajectories.

## Conclusion

This work suggests avoiding the usual LMM analysis of longitudinal HRQoL data in cancer clinical trials where patients may drop out. In general, dropout is associated with poor rather than good HRQoL, so that the LMM will be too optimistic in estimating the HRQoL parameters. In particular, the LMM will overestimate the slope governing the HRQoL trajectory in both arms. The direct and indirect effects of the treatment on the risk of dropout act together, potentially in opposite directions, but in general, the overall treatment effect is protective; that is, time before dropout is longer in the experimental arm. If this is the case, the LMM will also misestimate the effect of the treatment over time on HRQoL (i.e., the slope difference between experimental and control arms). More specifically, the LMM will be more optimistic for the control arm than for the experimental arm: where there is better HRQoL in the experimental arm, the LMM will diminish the beneficial treatment effect on HRQoL, and where there is poorer HRQoL in the experimental arm, it will accentuate the deleterious treatment effect on HRQoL. Such biases can be avoided using a JM and, as the JM is composed of a linear mixed submodel, the way of interpreting the HRQoL parameter results will be unchanged. On top of that, as the JM is also composed of a survival submodel, the JM will provide additional estimates and give further insight into the relationship between dropout and the longitudinal HRQoL outcome.

## Supplementary Information

**Additional file 1: Supplementary Table 1.** Complementary results to Table 1 regarding parameters *β*_0_, *σ*, *σ*_0_, *σ*_1_, *ρ*_01_. **Supplementary Table 2.** Main results of the JM (assuming a Weibull distribution for the baseline hazard function) fitted to the clinical trial data. **Supplementary Table 3.** Mean values of the median survival time in the 1000 simulations of the simulation study in each arm and in total. **Supplementary Table 4.** Complementary results to Table 2 regarding parameter *β*_0_, *σ*, *σ*_0_, *σ*_1_, *ρ*_01_.

## Data Availability

The dataset from the PRODIGE 4/ACCORD 11 clinical trial is not publicly available due to confidentiality requirements. Data are however available from the main coordinator of the clinical trial, Pr Thierry Conroy, upon reasonable request, and with permissions of the study sponsor UNICANCER R&D. The R scripts to perform the data analysis and to reproduce the simulation study are available from the authors upon request.

## Abbreviations

HRQoL: Health-related quality of life
LMM: Linear mixed model
PRO: Patient-reported outcome
JM: Joint model
HR: Hazard ratio
EORTC: European Organization for Research and Treatment of Cancer
ITT: Intention-to-treat
CI: Confidence interval
RMSE: Root mean square error
RB: Relative bias
Cov.: Coverage rate
