# Supplementary material for: When a joint model should be preferred over a linear mixed model for analysis of longitudinal health-related quality of life data in cancer clinical trials
Source: BMC Med Res Methodol. 2023 Feb 10;23:36. doi: 10.1186/s12874-023-01846-3 (PMC9912607; doi:10.1186/s12874-023-01846-3)
Supplement: Supplementary file 1 — Additional file 1: Supplementary Table 1. Complementary results to Table 1 regarding parameters β0, σ, σ0, σ1, ρ01. Supplementary Table 2. Main results of the JM (assuming a Weibull distribution for the baseline hazard function) fitted to the clinical trial data. Supplementary Table 3. Mean values of the median survival time in the 1000 simulations of the simulation study in each arm and in total. Supplementary Table 4. Complementary results to Table 2 regarding parameter β0, σ, σ0, σ1, ρ01. [file 12874_2023_1846_MOESM1_ESM.docx]

**File name:** Supporting_Information

**File format: .**docx

**Description of data:**

- Supplementary Table 1: Complementary results to Table 1 regarding parameters $\beta_{0}$, $\sigma$, $\sigma_{0}$, $\sigma_{1}$, $\rho_{01}$
- Supplementary Table 2: Main results of the JM (assuming a Weibull distribution for the baseline hazard function) fitted to the clinical trial data
- Supplementary Table 3: Mean values of the median survival time in the 1000 simulations of the simulation study in each arm (control and experimental) and in total
- Supplementary Table 4: Complementary results to Table 2 regarding parameter $\beta_{0}$, $\sigma$, $\sigma_{0}$, $\sigma_{1}$, $\rho_{01}$

|  | LMM | | | | | JM | | | | |
| --- | --- | --- | --- | --- | --- | --- | --- | --- | --- | --- |
|  | $\hat{\beta}_{0}$ [95% CI] | $\hat{\sigma}$ [95% CI] | $\hat{\sigma}_{0}$ [95% CI] | $\hat{\sigma}_{1}$ [95% CI] | $\hat{\rho}_{01}$* [95% CI] | $\hat{\beta}_{0}$ [95% CI] | $\hat{\sigma}$ [95% CI] | $\hat{\sigma}_{0}$ [95% CI] | $\hat{\sigma}_{1}$ [95% CI] | $\hat{\rho}_{01}$ [95% CI] |
| GLOBAL HEALTH STATUS/HRQoL |  |  |  |  |  |  |  |  |  |  |
| Global health status/HRQoL | 53.70  [51.75;55.65] | 13.42  [12.94;13.92] | 15.46  [13.92;17.18] | 2.50  [1.93;3.24] | -0.47  [-0.62;-0.28] | 53.84  [51.93;55.76] | 13.55  [13.06;14.06] | 15.08  [13.58;32.12] | 2.03  [1.51;2.73] | -0.36  [-0.56;-0.12] |
| FUNCTIONAL SCALES |  |  |  |  |  |  |  |  |  |  |
| Physical functioning | 74.55  [72.25;76.85] | 12.37  [11.91;12.85] | 19.55  [17.84; 21.41] | 2.76  [2.13;3.58] | -0.32  [-0.51;-0.09] | 74.69  [72.38;77] | 12.38  [72.38;77] | 19.63  [17.94;39.1] | 2.71  [2.05;3.58] | -0.28  [-0.52;-0.01] |
| Role functioning | 58.68  [55.31;62.05] | 18.96  [18.28;19.66] | 28.42  [25.88;31.20] | 3.96  [3.17;4.94] | -0.54  [-0.68;-0.36] | 58.93  [55.58;62.27] | 18.99  [18.31;19.69] | 28.17  [25.67;46.77] | 3.69  [2.89;4.7] | -0.47  [-0.71;-0.13] |
| Social functioning | 67.63  [64.57;70.70] | 16.73  [16.13;17.36] | 25.95  [23.67;28.46] | 3.37  [2.67;4.25] | -0.50  [-0.65;-0.32] | 67.82  [64.77;70.88] | 16.67  [16.08;17.29] | 25.86  [23.6;42.95] | 3.44  [2.72;4.35] | -0.44  [-0.67;-0.12] |
| SYMPTOM SCALES |  |  |  |  |  |  |  |  |  |  |
| Fatigue | 49.72  [47.06;52.39] | 16.72  [16.13;17.34] | 21.86  [19.82;24.11] | 3.56  [2.85;4.44] | -0.46  [-0.61;0.28] | 49.5  [46.87;52.13] | 16.8  [16.2;17.43] | 21.52  [19.53;34.96] | 3.18  [2.48;4.06] | -0.72  [-1.90; 0.47] |
| Pain | 36.85  [34.25;39.45] | 19.87  [19.17;20.59] | 19.96  [17.82; 22.37] | 3.47  [2.67;4.52] | -0.61  [-0.74;-0.43] | 36.46  [33.9;39.02] | 20.05  [19.33;20.8] | 19.33  [17.23;30.46] | 2.59  [1.82;3.7] | -0.49  [-0.75;-0.11] |

**Supplementary Table 1.** Complementary results of the LMM and JM fitted to the clinical trial data

*Estimate of the correlation parameter defined as $\rho_{01}=\sigma_{01}/\sigma_{0}\sigma_{1}$

JM, joint model; LMM, linear mixed model: CI, confidence interval

**Supplementary Table 2.** Main results of the JM (assuming a Weibull distribution for the baseline hazard function) fitted to the clinical trial data

|  | HRQoL score trajectory | | | | Risk of dropout | | | |
| --- | --- | --- | --- | --- | --- | --- | --- | --- |
|  | Time effect | | Arm-by-time interaction effect | | Arm effect | | Association with HRQoL | |
|  | $\hat{\beta}_{1}$ [95% CI] | $p$ | $\hat{\beta}_{2}$ [95% CI] | $p$ | $\hat{\gamma}_{1}$ [95% CI] | $p$ | $\hat{\alpha}$ [95% CI] | $p$ |
| GLOBAL HEALTH STATUS/HRQoL |  |  |  |  |  |  |  |  |
| Global health status/HRQoL | 0.36  [-0.41; 1.13] | 0.357 | 1.17  [0.31; 2.04] | **0.008** | -0.42  [-0.69; -0.15] | **0.003** | -0.022  [-0.031; -0.012] | <$\mathbf{1}\mathbf{0}^{-\mathbf{4}}$ |
| FUNCTIONAL SCALES |  |  |  |  |  |  |  |  |
| Physical functioning | -0.66  [-1.55; 0.24] | 0.150 | 0.77  [-0.20; 1.74] | 0.118 | -0.62  [-0.89; -0.34] | <$\mathbf{1}\mathbf{0}^{-\mathbf{4}}$ | -0.026  [-0.034; -0.018] | <$\mathbf{1}\mathbf{0}^{-\mathbf{4}}$ |
| Role functioning | 0.36  [-0.82; 1.54] | 0.555 | 1.16  [-0.15; 2.48] | 0.083 | -0.53  [-0.80; -0.26] | <$\mathbf{1}\mathbf{0}^{-\mathbf{4}}$ | -0.016  [-0.022; -0.011] | <$\mathbf{1}\mathbf{0}^{-\mathbf{4}}$ |
| Social functioning | -0.28  [-1.36; 0.80] | 0.609 | 1.25  [0.03; 2.47] | **0.045** | -0.53  [-0.80; -0.26] | <$\mathbf{1}\mathbf{0}^{-\mathbf{4}}$ | -0.016  [-0.021; -0.011] | <$\mathbf{1}\mathbf{0}^{-\mathbf{4}}$ |
| SYMPTOM SCALES |  |  |  |  |  |  |  |  |
| Fatigue | -0.85  [-1.91; 0.20] | 0.113 | -0.65  [-1.84; 0.55] | 0.289 | -0.59  [-0.86; -0.32] | <$\mathbf{1}\mathbf{0}^{-\mathbf{4}}$ | 0.016  [0.010; 0.022] | <$\mathbf{1}\mathbf{0}^{-\mathbf{4}}$ |
| Pain | -2.07  [-3.11; -1.04] | <$\mathbf{1}\mathbf{0}^{-\mathbf{3}}$ | -0.90  [-2.04; 0.25] | 0.125 | -0.48  [-0.74; -0.21] | <$\mathbf{1}\mathbf{0}^{-\mathbf{3}}$ | 0.013  [0.006; 0.020] | <$\mathbf{1}\mathbf{0}^{-\mathbf{3}}$ |

HRQoL, health-related quality of life; JM, joint model; LMM, linear mixed model; CI, confidence interval

**Supplementary Table 3.** Mean values of the median survival time in the 1000 simulations

|  |  | **Median survival time (months)** |
| --- | --- | --- |
| **SCENARIO 0** |  |  |
|  | Control arm | 6.2 |
|  | Experimental arm | 8.7 |
|  | **Total** | **7.2** |
| **SCENARIO 1** |  |  |
|  | Control arm | 9.0 |
|  | Experimental arm | 9.0 |
|  | **Total** | **9.0** |
| **SCENARIO 2** |  |  |
|  | Control arm | 8.4 |
|  | Experimental arm | 10.0 |
|  | **Total** | **9.1** |
| **SCENARIO 3** |  |  |
|  | Control arm | 8.4 |
|  | Experimental arm | 17.0 |
|  | **Total** | **11.3** |
| **SCENARIO 4** |  |  |
|  | Control arm | 8.4 |
|  | Experimental arm | 13 |
|  | **Total** | **10.4** |
| **SCENARIO 5** |  |  |
|  | Control arm | 8.4 |
|  | Experimental arm | 10.8 |
|  | **Total** | **9.6** |

|  |  | **LMM** | | | | | **JM** | | | | |
| --- | --- | --- | --- | --- | --- | --- | --- | --- | --- | --- | --- |
|  | **True** | **Mean** | **Bias** | **RB** | **RMSE** | **Cov.** | **Mean** | **Bias** | **RB** | **RMSE** | **Cov.** |
| **SCENARIO 0** | | | | | | | | | | | |
| **HRQoL** | | | | | | | | | | | |
| $\beta_{0}$ | 53.9 | 53.576 | -0.324 | -0.006 | 0.829 | 93.3 | 53.91 | 0.010 | 0 | 0.765 | 96.1 |
| $\sigma$ | 13.5 | 13.517 | 0.017 | 0.001 | 0.232 | 95.1 | 13.504 | 0.004 | 0 | 0.230 | 95.5 |
| $\sigma_{0}$ | 15.2 | 15.18 | -0.02 | -0.001 | 0.646 | 94.6 | 15.169 | -0.031 | -0.002 | 0.647 | 94.6 |
| $\sigma_{1}$ | 2.1 | 2.082 | -0.018 | -0.008 | 0.141 | 94.6 | 2.087 | -0.013 | -0.006 | 0.139 | 93.7 |
| $\rho_{01}$ | -0.4 | -0.411 | -0.011 | 0.028 | 0.068 | 94 | -0.398 | 0.002 | -0.005 | 0.068 | 96.1 |
| **Risk of death** | | | | | | | | | | | |
| $\gamma_{0}$ | -2.2 | - | - | - | - | - | -2.207 | -0.007 | 0.003 | 0.173 | 94.7 |
| $\phi$ | 1.6 | - | - | - | - | - | 1.606 | 0.006 | 0.004 | 0.063 | 94.5 |
| **SCENARIO 1** | | | | | | | | | | | |
| **HRQoL** | | | | | | | | | | | |
| $\beta_{0}$ | 53.9 | 53.913 | 0.013 | 0 | 0.759 | 95.6 | 53.916 | 0.016 | 0 | 0.761 | 95.8 |
| $\sigma$ | 13.5 | 13.502 | 0.002 | 0 | 0.222 | 95.6 | 13.502 | 0.002 | 0 | 0.221 | 95.6 |
| $\sigma_{0}$ | 15.2 | 15.215 | 0.015 | 0.001 | 0.658 | 94.2 | 15.196 | -0.004 | 0 | 0.657 | 93.9 |
| $\sigma_{1}$ | 2.1 | 2.101 | 0.001 | 0.001 | 0.125 | 95.8 | 2.094 | -0.006 | -0.003 | 0.125 | 93.8 |
| $\rho_{01}$ | -0.4 | -0.4 | 0 | -0.001 | 0.061 | 95.3 | -0.4 | 0 | 0.001 | 0.062 | 96.0 |
| **Risk of death** | | | | | | | | | | | |
| $\gamma_{0}$ | -4.1 | - | - | - | - | - | -4.106 | -0.006 | 0.001 | 0.192 | 95.1 |
| $\phi$ | 1.7 | - | - | - | - | - | 1.705 | 0.005 | 0.003 | 0.062 | 95.4 |
| **SCENARIO 2** | | | | | | | | | | | |
| **HRQoL** | | | | | | | | | | | |
| $\beta_{0}$ | 53.9 | 53.434 | -0.466 | -0.009 | 0.903 | 91.9 | 53.888 | -0.012 | 0 | 0.774 | 94.7 |
| $\sigma$ | 13.5 | 13.533 | 0.033 | 0.002 | 0.222 | 94.8 | 13.506 | 0.006 | 0 | 0.218 | 95.4 |
| $\sigma_{0}$ | 15.2 | 15.195 | -0.005 | 0 | 0.614 | 95.6 | 15.183 | -0.017 | -0.001 | 0.616 | 95.4 |
| $\sigma_{1}$ | 2.1 | 2.062 | -0.038 | -0.018 | 0.124 | 94.8 | 2.09 | -0.01 | -0.005 | 0.117 | 95.3 |
| $\rho_{01}$ | -0.4 | -0.426 | -0.026 | 0.065 | 0.064 | 93.2 | -0.401 | -0.001 | 0.002 | 0.060 | 95.2 |
| **Risk of death** | | | | | | | | | | | |
| $\gamma_{0}$ | -2.2 | - | - | - | - | - | -2.21 | -0.01 | 0.005 | 0.186 | 95.3 |
| $\phi$ | 1.6 | - | - | - | - | - | 1.606 | 0.006 | 0.004 | 0.065 | 94.5 |
| **SCENARIO 3** | | | | | | | | | | | |
| **HRQoL** | | | | | | | | | | | |
| $\beta_{0}$ | 53.9 | 53.511 | -0.389 | -0.007 | 0.85 | 92.3 | 53.915 | 0.015 | 0 | 0.754 | 95.6 |
| $\sigma$ | 13.5 | 13.528 | 0.028 | 0.002 | 0.215 | 93.8 | 13.505 | 0.005 | 0 | 0.212 | 94.4 |
| $\sigma_{0}$ | 15.2 | 15.214 | 0.014 | 0.001 | 0.611 | 95.3 | 15.194 | -0.006 | 0 | 0.612 | 95.5 |
| $\sigma_{1}$ | 2.1 | 2.063 | -0.037 | -0.018 | 0.114 | 93.8 | 2.092 | -0.008 | -0.004 | 0.108 | 94.4 |
| $\rho_{01}$ | -0.4 | -0.421 | -0.021 | 0.053 | 0.06 | 92.1 | -0.401 | -0.001 | 0.002 | 0.056 | 95.6 |
| **Risk of death** | | | | | | | | | | | |
| $\gamma_{0}$ | -2.2 | - | - | - | - | - | -2.214 | -0.014 | 0.006 | 0.21 | 94.5 |
| $\phi$ | 1.6 | - | - | - | - | - | 1.608 | 0.008 | 0.005 | 0.07 | 94.4 |
| **SCENARIO 5** | | | | | | | | | | | |
| **HRQoL** | | | | | | | | | | | |
| $\beta_{0}$ | 53.9 | 53.454 | -0.446 | -0.008 | 0.875 | 91.6 | 53.935 | 0.035 | 0.001 | 0.754 | 95.4 |
| $\sigma$ | 13.5 | 13.523 | 0.023 | 0.002 | 0.215 | 95.2 | 13.501 | 0.001 | 0 | 0.213 | 95.1 |
| $\sigma_{0}$ | 15.2 | 15.207 | 0.007 | 0 | 0.632 | 95.4 | 15.187 | -0.013 | -0.001 | 0.632 | 94.9 |
| $\sigma_{1}$ | 2.1 | 2.072 | -0.028 | -0.013 | 0.122 | 94.6 | 2.092 | -0.008 | -0.004 | 0.118 | 93.8 |
| $\rho_{01}$ | -0.4 | -0.421 | -0.021 | 0.053 | 0.063 | 92.7 | -0.398 | 0.002 | -0.005 | 0.061 | 95.4 |
| **Risk of death** | | | | | | | | | | | |
| $\gamma_{0}$ | -2.2 | - | - | - | - | - | -2.207 | -0.007 | 0.003 | 0.173 | 94.7 |
| $\phi$ | 1.6 | - | - | - | - | - | 1.606 | 0.006 | 0.004 | 0.063 | 94.5 |

**Supplementary Table 4.** Results^a^ of the simulation study on $\beta_{0}$ and the variance and Weibull parameters

^a^ mean, bias, relative bias (RB), root mean square error (RMSE) and coverage rate (Cov.) based on 1000 generated datasets of 500 patients

HRQoL, health-related quality of life; JM, joint model; LMM, linear mixed model
